# Film-thickness-driven superconductor to insulator transition in cuprate superconductors

**DOI:** 10.1038/s41598-020-60037-y

**Published:** 2020-02-24

**Authors:** Han-Byul Jang, Ji Soo Lim, Chan-Ho Yang

**Affiliations:** 1 Department of Physics, KAIST, Daejeon, 34141 Republic of Korea; 2 Center for Lattice Defectronics, KAIST, Daejeon, 34141 Republic of Korea; 3 KAIST Institute for the NanoCentury, KAIST, Daejeon, 34141 Republic of Korea

**Keywords:** Superconducting properties and materials, Electronic properties and materials

## Abstract

The superconductor-insulator transition induced by film thickness control is investigated for the optimally doped cuprate superconductor La_1.85_Sr_0.15_CuO_4_. Epitaxial thin films are grown on an almost exactly matched substrate LaAlO_3_ (001). Despite the wide thickness range of 6 nm to 300 nm, all films are grown coherently without significant relaxation of the misfit strain. Electronic transport measurement exhibits systematic suppression of the superconducting phase by reducing the film thickness, thereby inducing a superconductor-insulator transition at a critical thickness of ~10 nm. The emergence of a resistance peak preceding the superconducting transition is discussed based on the weak localization. X-ray photoelectron spectroscopy results show the possibility that oxygen vacancies are present near the interface.

## Introduction

The superconductor-to-insulator transition (SIT) can be induced by control of magnetic vortex density^1^ and charge carrier density^2–8^. Effective dimension control, e.g. variation of film thickness, can be an alternative pathway to derive the SIT in superconducting layers^9–13^. Despite many identifications of SIT^1–13^, the exact origin has not yet been fully understood. In addition to the film thickness what one intends to control, many other intrinsic and/or extrinsic factors can be involved in significant modifications of electronic conduction^9–13^. In particular, unconventional cuprate superconductors are vulnerable to disorder due to the presence of nodes in the superconducting gap^14–16^. To elucidate the main factors which determine electronic conduction properties, it is necessary to scrutinize the crystalline and electronic structures as well as electronic conduction properties comprehensively. In this paper, we focus on thickness-controlled epitaxial layers for the optimally doped cuprate superconductor.

La_2−x_Sr_x_CuO_4_ (LSCO) is an unconventional high *T*_c_ superconducting system. It shows a superconducting ground state in the Sr doping range of 0.07 < x < 0.22 in bulk^17^ with a maximum critical temperature (*T*_c_) of ~40 K at x ~ 0.15. When the doping ratio is 1/8, a stripe phase with spatial inhomogeneity in charge and spin densities emerges^18,19^, suppressing superconductivity, thereby forming a plateau in the superconducting dome^17,20^. Excess or lack of oxygen ions also influences the electronic structure and the superconducting state^21–23^. The LSCO is a layered perovskite structure which has two competing high-temperature tetragonal and low-temperature orthorhombic phases. Its structural phase transition temperature quickly decreases with increasing Sr doping around x ~ 0.2^24,25^.

It has been reported that epitaxial thin films of the cuprates can have a significantly modified *T*_c_ due to compressive or tensile misfit strains from substrates^12,13,20,22,26,27^. The compressive strain enhances the *T*_c_ with elongation of the *c*-axis lattice parameter, whereas tensile strain reduces the *T*_c_^22,26,27^. Although most studies were carried out based on the popular substrates of SrTiO_3_ (STO) and LaSrAlO_4_ (LSAO), it has been revealed that the misfit strains are rapidly relaxed with increasing film thickness. This strain relaxation problem hindered direct exploration of film thickness effects without significant changes in lattice parameters. As a result, this problem has prevented us from reaching an agreement on the origins of the SITs.

In this study, to minimize the strain relaxation issue, we use LaAlO_3_ (LAO) substrates which offer the most similar in-plane lattice parameter with that of the bulk LSCO at x = 0.15, among commercially available oxide substrates. A series of thin films are deposited with varying film thickness from 6 nm to ~300 nm. We find that film-thickness-driven SIT occurs at ~10 nm with a resistance peak near superconducting *T*_c_ possibly due to the weak localization. The finding is also discussed in the context of a presence of oxygen vacancies near the interface.

## Results

### Thickness-driven superconductor to insulator transition

**Figure 1:**
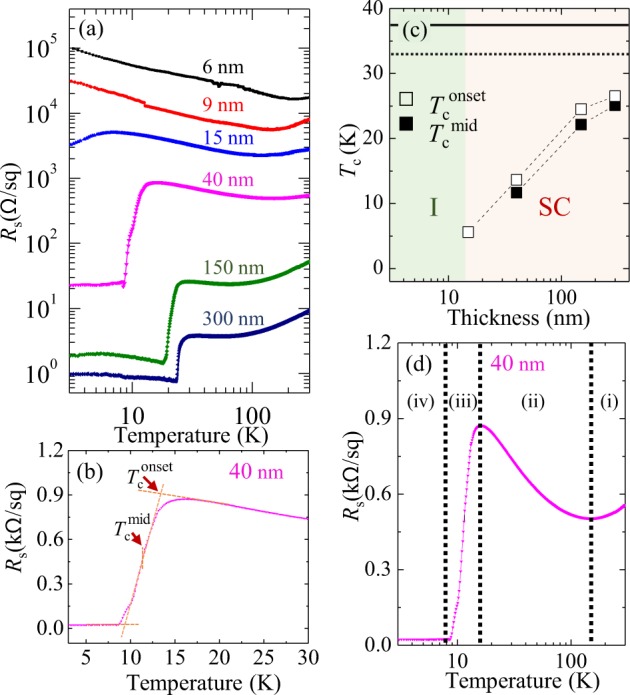
(**a**) Temperature dependence of sheet resistance *R*_s_ for La_1.85_Sr_0.15_CuO_4_ thin films with film thicknesses of 6, 9, 15, 40, ~150 and ~300 nm. (**b**) The magnified linear-scale *R*_s_ − *T* curve of the 40-nm-thick film. The onset superconducting transition temperature is defined by the crossing point of two yellow extrapolated lines. The midpoint temperature is defined as the temperature at which resistance becomes half of the resistance at the onset temperature relative to the base resistance. (**c**) Superconducting transition temperatures versus logarithmic film thickness. The horizontal solid (dashed) line indicates the onset (midpoint) transition of the bulk target. (**d**) Semi-logarithmic *R*_s_ − *T* plot for the 40-nm-thick film. The labels of (i) to (iv) represent four conduction regimes that correspond to the metal, insulator, transition, and superconductor.

Figure 1(a) shows temperature dependence of the sheet resistance *R*_s_ for various film thicknesses. The series of epitaxial films show a clear film-thickness-driven SIT at a critical thickness of ~10 nm. The films thinner than or equal to 9 nm exhibit an insulating behavior in which the resistance is linearly increasing (expected to follow a power law) with cooling in the double-logarithmic plot over the whole measuring temperatures except for an upturn near room temperature. The signature of the superconducting transition emerges at the film thickness of 15 nm showing a downturn of resistance below 10 K, while the thicker samples exhibit more clear superconducting transitions. Despite of the superconducting transition, resistance is not perfectly zero but exhibits a sensibly large value that increases with decreasing film thickness. A spatial inhomogeneity disturbs the coherence of the superconducting order parameter leading to a resistance increase due to phase fluctuation^28–30^. We also note granular systems show charging effects which can lead to similar manifestations^31^. Fermionic dissipation in the presence of coupling with a bath has been discussed in similar contexts^32^.

For a more quantitative comparison, we define the superconducting transition temperatures, *T*_c_^onset^ and *T*_c_^mid^, as described in Fig. 1(b). Figure 1(c) presents *T*_c_^onset^ and *T*_c_^mid^ as a function of logarithmic film thickness. The superconducting transition temperature increases monotonically with film thickness, but *T*_c_^onset^ for the thickest film is still lower than the value (37 K) of our bulk target used for film growth. We also observe that the superconducting transition is not abrupt but exhibits a gradual variation with a finite temperature width as much as ~5 K, suggesting the presence of disorder.

Furthermore, we can observe a resistance peak preceding the onset of the superconductivity (Fig. 1(d)). The peak arises at a high temperature of ~150 K and exists over a wide range of temperatures. It is worthwhile mentioning that the similar resistance upturn preceding the superconducting transition was observed in under-doped cuprates and explained on the basis of the weak localization^33^ and the Maki-Thompson superconducting fluctuation^34^. A theoretical effort was made to explain the upturn by comprehensively combining the Aslamazov-Larkin and Maki-Thomson fluctuations, weak localization, and the reduction of the density of states near the superconducting transition^35^. It has also been reported that the similar resistance peak can be produced by an inclination of the CuO_2_ planes^36^ (that can be ruled out by a negligible mosaic tilting angle characterized by x-ray diffraction) and strong localization of bosonic particles^37^.

Comparisons of length scales will be useful for a better understanding of the mechanism in this system. The intrinsic superconducting coherence length (the size of a Cooper pair) is ${\xi }_{0}=\hslash {v}_{F}/\pi \Delta$ in the framework of the BCS theory. The length in cuprates was estimated to be 15–20 Å (4–5 unit cells) taking into account an important statistical weight at the singular points corresponding to zero Fermi velocity (*v*_F_ = 0)^38^. The stronger the binding energy (Δ), the smaller the size (ξ_0_) of a single Cooper pair. If impurities are too dense and exist within the length scale, the pairing can be destroyed. Once Cooper pairs form as quasiparticles in a cleaner condition, we can next consider phase coherence among the Cooper pairs. The phase coherence length of the superconducting order parameter, which represents the typical size of a superconducting domain where a coherent Bose-Einstein condensate occurs, can be much larger than the intrinsic size. The electron mean free path was estimated to be ~700 Å in the clean-limit regime^39^ and it can be reduced by the quenched disorder potential producing residual resistivity. The structural coherence length limited by film thickness and/or grain size along lateral directions is also another important length scale to be considered. We note that the samples of which the thicknesses are much smaller than the phase coherence length can be regarded to be in the two-dimensional transport limit.

For explaining the resistance peak preceding the superconducting transition as well as the residual resistance, we might introduce a percolation concept which can be important when the phase coherence length is significantly smaller than the device size. But, our experiments cannot explicitly quantify the length at the present time, despite hints from the granular features in surface morphology discussed later. Since the de Broglie’s wavelength of a fermionic electron is also ~1 nm (smaller than the mean free path) at the Fermi velocity ~0.7 × 10^8^ cm/s, one can interpret the resistance peak on the basis of the weak localization that results in a seemingly insulating feature.

### Possible weak localization

**Figure 2:**
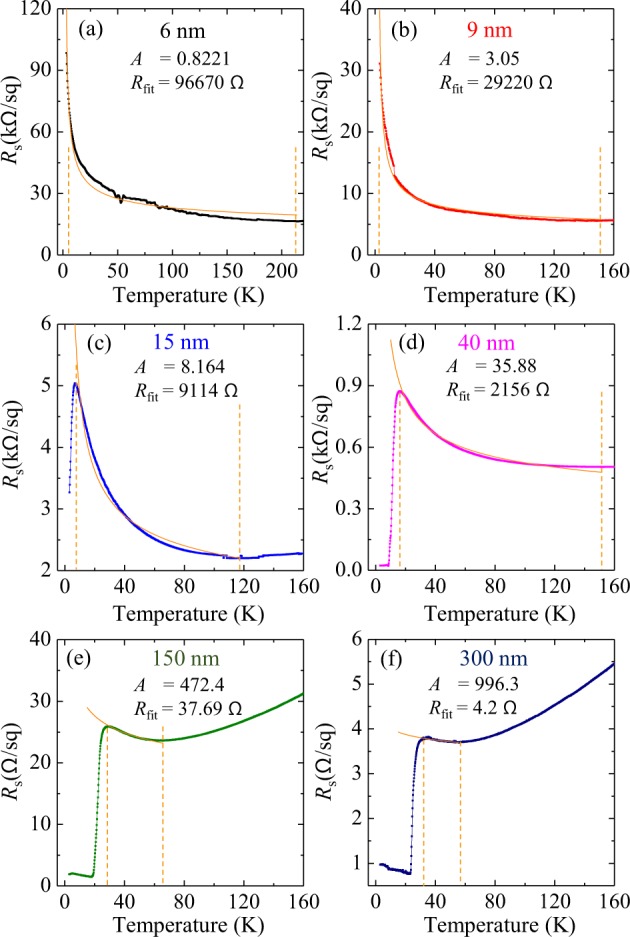
Theoretical fitting to the resistance peak for (**a**) 6 nm (**b**) 9 nm (**c**) 15 nm (**d**) 40 nm (**e**) ~150 nm and (**f**) ~300 nm thick films. The parameters *A* and *R*_fit_ were determined for each film thickness by fitting the theoretical curve (orange solid line) associated with the weak localization and interaction in a diffusion channel to the experimental data (square symbols) in the temperature range indicated by the two vertical dashed lines.

It is interesting to explore whether the weak localization with the correction terms of superconducting fluctuations can explain the observed resistance peak. According to the previous study^35^, the superconducting fluctuation corrections (regarding the Aslamazo-Larkin, Maki-Thompson, and the reduction of density of states) compensate each other above *T*_c_. Hence, we analyze the insulating region of the observed resistance peak above *T*_c_ by using the combination of the weak localization term (Δ*G*^*WL*^) and a correction term regarding the interaction in a diffusion channel (Δ*G*^*ID*^), which is given by:

1 $$\frac{\Delta {G}^{WL}(T)+\Delta {G}^{ID}(T)}{{G}_{00}}=A\cdot \,\mathrm{ln}[\frac{{k}_{B}T\tau }{\hslash }]$$

where *G*_00_ = *e*^2^/(2π^2^*ℏ*), *τ* is relaxation time (2 ps), and *A* is a proportional coefficient. The alignment of the theoretical curves and the experimental data was carried out by using the formula:

2 $$R(T)=\frac{1}{(\Delta {G}^{WL}(T)+\Delta {G}^{ID}(T))+1/{R}_{fit}}$$

where *R*_*fit*_ and the coefficient *A* in Eq. (1) are fitting parameters. Figure 2 shows linearly scaled *R*_s_ − *T* curves with the theoretical fitting lines. The fitting range was taken to be between the temperature giving the maximum of a resistance peak and the minimum of an upturn curvature. The fitting curves show reasonably good matching with the experimental data, suggesting the weak localization is a plausible mechanism of the resistance peak.

Nonetheless, it is worth mentioning that a power law behavior may alternatively explain the curvatures of *R*_s_ − *T* curves. We perform fitting the curves into a power law formula to determine the exponent; −0.41 for 6 nm, −0.47 for 9 nm, −0.34 for 15 nm. For the thinnest (6 nm) sample, the power law behavior is more consistent with the experimental curve than the localization formula. But the thicker ones show better matches with the weak localization formula. It has been reported that the electron-electron interaction can result in *R*_s_ ~ 1/√*T* (ref. ^40^). The determined exponent (−0.41) at the thinnest sample is not far from −0.5 but is clearly distinguished. As the thickness increases, the absolute value of the exponent tends to decrease (see the linear slopes in the double-logarithmic plots in Fig. 1(a)).

### Crystalline structural analysis

**Figure 3:**
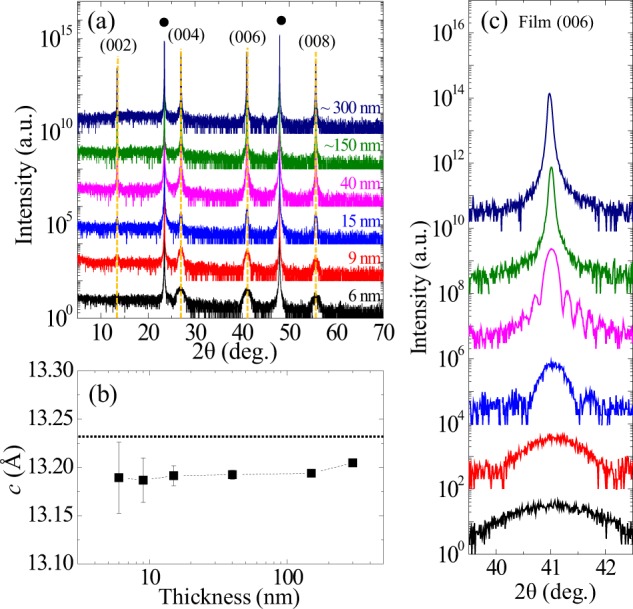
Crystal structural characterization by x-ray diffraction. (**a**) X-ray 2θ − ω scans for La_1.85_Sr_0.15_CuO_4_ thin films on LaAlO_3_ substrate. Black circles represent the peaks of LaAlO_3_ substrate. Vertical orange dashed lines exhibit the peak positions of the La_1.85_Sr_0.15_CuO_4_ films were almost identical regardless of different film thicknesses. (**b**) *c*-axis lattice parameter versus logarithmic film thickness. Error bar was evaluated from the FWHM of (006) peak. (**c**) The enlarged 2θ − ω scans for (006) peaks. The thicknesses of films thinner than 100 nm were defined by the Kiessig fringes and/or the Scherrer formula, while the two thickest films were estimated by growth time.

To investigate the existence of thickness-driven structural effects such as strain relaxation, we measured x-ray 2θ − ω scans for the series of samples (Fig. 3(a,b)). Only the LSCO (00L) peaks except for the LAO substrate peaks are observed indicating the films are highly oriented without any noticeable secondary or impurity phases. Even if the maximum film thickness reaches ~300 nm, the whole films exhibit almost identical *c*-axis lattice parameters, within the error bar of less than 0.2%. No significant strain relaxation was observed in our films because we used LAO substrate of which the in-plane lattice parameter that is very close to the bulk LSCO lattice parameter (*a*_LAO_ = 3.789 Å and *a*_LSCO_ = 3.777 Å^25^). As compared with the layered perovskite LSAO and the perovskite STO (*a*_LSAO_ = 3.754 Å and *a*_STO_ = 3.905 Å), those have been popular substrates for the study of superconducting LSCO films with providing misfit strains of −0.61% and +3.39% to the LSCO film, the LAO substrate has an advantage of a better lattice mismatch despite the concern of the rhombic unit. Regardless of the good lattice parameter match between film and substrate, the *c*-axis lattice parameters of the films are still observed to be comparatively smaller than the bulk value due to the noticeable tensile strain (+0.32%) from LAO substrate. As shown in the Fig. 3(c), we can clearly identify the Kiessig fringes around the film diffraction peaks due to the well-defined uniform film thickness. However, since it was difficult to observe the expected fast thickness oscillations in the two thickest samples, the film thickness was predicted using the deposition time.

**Figure 4:**
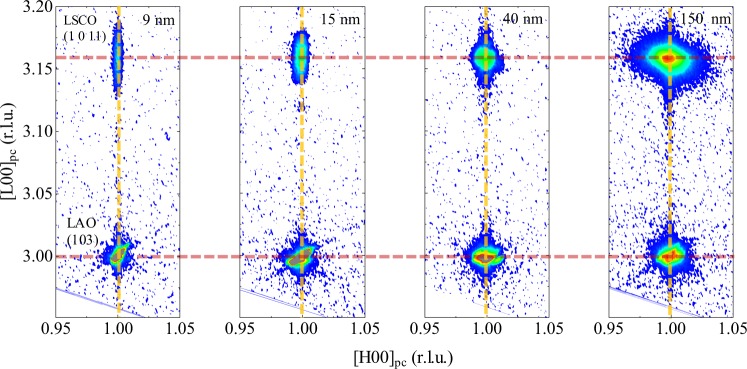
Reciprocal space maps of (1 0 11) film peak near (103) substrate peak for four different thick films. Reciprocal lattice unit (r.l.u.) is defined as 2π divided by the pseudocubic lattice parameter (3.789 Å) of LaAlO_3_ substrate. In-plane lattice parameters of the films exactly match with those of substrates, indicating fully strained films regardless of the different thicknesses. Out-of-plane lattice parameters are also almost same as a result of no significant strain relaxation.

To further examine the strain state of the films, we conducted reciprocal space mapping (RSM) for the (1 0 11) LSCO film peak with (103) LAO substrate peak. Figure 4 shows reciprocal space maps of the 9, 15, 40, and ~150-nm-thick films. The in-plane reciprocal positions of the film peaks are exactly matched with the substrate, indicating that the films are fully strained to substrates irrespective of the different film thicknesses. Since the (1 0 11) film peaks don’t result in any sensible peak splitting (e.g., along (00L) direction by monoclinic tilting), the crystal structure of film is assigned a tetragonal phase at room temperature, which is consistent with the bulk result^25^. LSCO films on LAO substrate obviously have high resistibility for strain relaxation due to the small misfit strain (+0.32%). For the biaxial-strained epitaxial film, the ratio of strains between out-of-plane and in-plane directions is 2*ν*/(1 − *v*), where *ν* is the Poisson ratio. From the structural characterization, we can obtain the average Poisson ratio of the films, *ν* = 0.294, which shows good agreement with the bulk value 0.3^41^. This structural analysis suggests that our films don’t have a significant strain relaxation that triggers the thickness-driven SIT.

The thickest (~150 nm) sample has a strong intensity of the (1 0 11) peak which enables us to evaluate the detailed shape of the peak in terms of the width and anisotropy. The peak looks diffuse along the mosaic rotation angle, but the full-width-at-half-maximum (FWHM) along the axis is still tiny (~0.1 degree), indicating the grains are aligned within the angle. The width along the horizontal H-axis is 0.0019 reciprocal lattice unit (r.l.u.). It guarantees that the structural coherence length along the horizontal axis in real space is at least more than 500 unit cells, which is estimated conservatively in terms of the fact that the resolution of the instrument gives a convolution effect.

### Granular features in surface morphology

**Figure 5:**
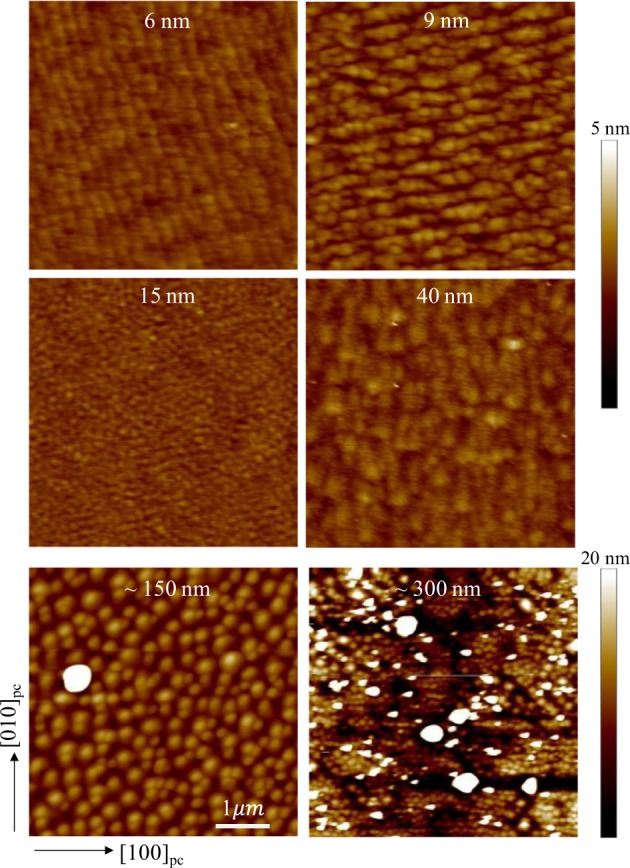
Surface topographic images for the thickness-series of samples. Granularity is observed in all the samples. The typical grain size doesn’t show a correlation with film thickness. The two thickest samples (~150 nm and ~300 nm) exhibit significantly higher surface roughness than the others.

Surface topographic images of the deposited epitaxial thin films were measured by atomic force microscopy. From the surface topographic images in Fig. 5, we were able to identify surface roughness and growth mode of the films. The 6-nm-thick film shows a well-defined step-terrace structure with flat surface. Topographic features of island growth mode begin to appear in thicker samples, however, the symptoms of step-terrace growth still remain up to 40-nm film thickness with relatively small surface roughness. For the thicker ~150 nm and ~300 nm films, the surface roughness of the film significantly increases up to the ~20 nm scale. Island growth mode is dominant in both films, whereas the features of the step-terrace structure are completely eliminated. Ironically, the poor surface quality in the thicker samples results in the relatively well-defined superconducting properties with higher *T*_c_.

Generally, the disorder effect arises in granular films, in which electronic conduction between neighboring grains is established either by Josephson tunneling via weak links or by quasiparticle tunneling^10^. Hence, the density of grain boundaries may serve as an important parameter for electronic conduction in granular systems. Especially, this granular effect becomes more crucial in ultrathin films because of low-dimensional networks of superconducting grains. Potential barriers across the boundary capacitors between superconducting grains may create the residual resistances. Typically, the grain size grows as the film thickness increases while the density of grain boundaries reduces. Thus, a positive correlation between electronic conduction and film thickness is anticipated. However, the LSCO thin films do not strictly follow the relationship between grain size and thickness, in contrast to the systematic change in electronic transport. Compared to the 9 and 150 nm films, the 15 and 300 nm films exhibit rather smaller grain sizes. From the surface topographic images, we expect the grain size in the lateral direction to be about 0.1~1 μm. This length scale is an order of magnitude longer than the mean free path of the LSCO thin films discussed in the electronic transport part. Comparison of the expected grain size with the mean free path indicates that changes in grain boundary density are not responsible for changes in electronic conduction and superconductivity. It suggests that other unidentified microscopic defects, such as oxygen deficiency, might cause disorder effects on the films.

### Cross-sectional transmission electron microscopy

**Figure 6:**
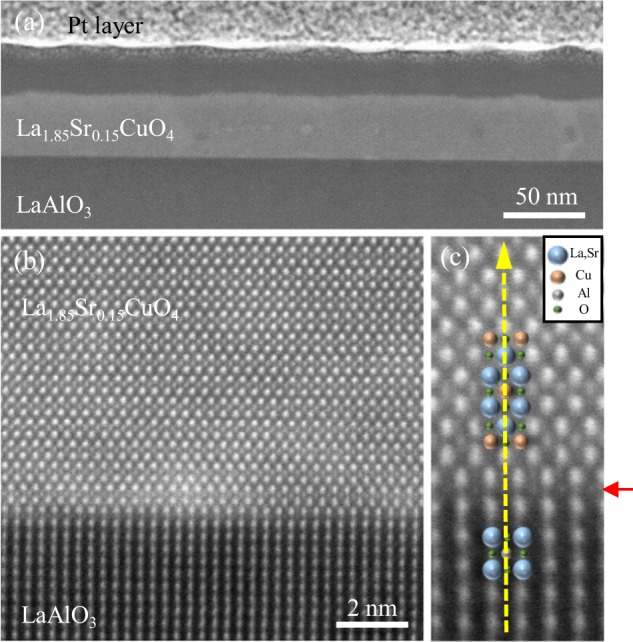
High-angle annular dark-field (HAADF) image of a La_1.85_Sr_0.15_CuO_4_ thin film. (**a**) A large area image of the film and substrate. (**b**) High-resolution image of the interfacial area between the thin film and the substrate. (**c**) The epitaxial relation between film and substrate at the interface. It can be also clearly seen that the thin film has a layered perovskite structure.

To gain more insight into the depth profile of the epitaxial thin film, we carried out transmission electron microscopy (TEM) for a cross-sectional view of a LSCO thin film. A low-magnification dark-field TEM image in Fig. 6(a) shows that the film is grown smoothly without any significant granular feature and severe inter-diffusion between film and substrate. To investigate the atomic structure and the epitaxial relationship between the film and the substrate, we obtained a high-angle annular dark-field (HAADF) image at an interfacial region (Fig. 6(b,c)). We were able to identify the layered perovskite structure at the atomic level and confirm the film was coherently grown on the LAO substrate. In the Z-contrast image, the heavier element reveals the brighter contrast. The A-site ions are clearly seen, however, light elements such as oxygen and aluminum ions are almost undetected. Accordingly, the B-site positions, where Al ions are located, in the substrate region were dark, while the Cu ions were relatively clearly detected in the film region. We note that the topmost layer of the substrate (as indicated by the red horizontal arrow) has a barely detectable contrast in the B-site positions suggesting Cu ions are mixing with Al ions in the interfacial layer. The first well-defined CuO_2_ layer was emergent with shifting half a unit cell along the horizontal axis, while the next CuO_2_ layer was right on the vertical axis (as represented by the yellow dashed arrow). This horizontal half-a-unit-cell shift between neighboring CuO_2_ layers alternatingly appears forming the layered perovskite. In addition, we performed energy dispersive spectroscopy (EDS) over the film area shown in Fig. 6(b) to check the atomic composition of the film. The atomic ratio of Sr to La was measured to be 0.0815. This ratio can be converted to Sr substitution ratio of 0.1507 in accord with the original stoichiometric value of the PLD target (La_1.85_Sr_0.15_CuO_4-δ_). We couldn’t observe dislocations and stacking faults in the high-resolution images indicating reasonably good crystallinity. However, we cannot still exclude the involvement of point defects such as oxygen vacancies.

### X-ray photoelectron spectroscopy analysis

**Figure 7:**
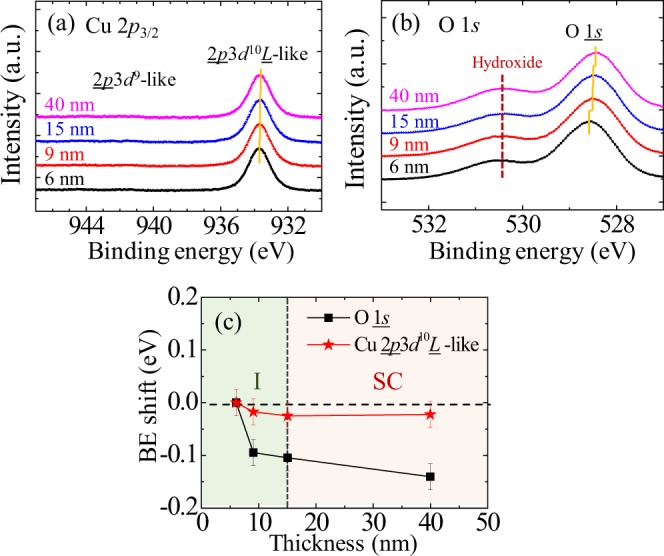
Core level XPS spectrum for 6, 9, 15, and 40-nm-thick films with 0.05 eV step size. (**a**) Core level Cu 2*p*_3/2_ XPS spectra. The main and suppressed satellite peaks correspond to 2*p*3*d*^10^*L*-like and 2*p*3*d*^9^-like states, respectively. Yellow guide line indicates the peak centers of 2*p*3*d*^10^*L*-like states. (**b**) O 1 *s* core XPS spectra. The yellow guide lines indicate the peak centers of O 1 *s* peaks. Wine dashed line indicates the surface hydroxide peaks. (**c**) Binding energy shifts of O 1 *s* and Cu 2*p*_3/2_ core levels relative to those of the 6-nm-thick film. Error bar represents a measurement step of ±0.025 eV.

We obtained core level x-ray photoelectron spectroscopy (XPS) for Cu 2*p*_3/2_ and O 1 *s* level to get information of the electronic structure for the 6, 9, 15, and 40-nm-thick films. The Cu 2*p*_3/2_ spectrum shown in Fig. 7(a) reveals a peak at 933.5 eV that corresponds to the binding energy of 2*p*3*d*^10^*L*–like state. No signature of the 2*p*3*d*^9^-like state, which is expected to give a peak at a higher binding energy of ~942 eV, suggests holes in our LSCO films prefer to reside on neighboring oxygen ligands rather than right on Cu *d*-orbitals^42–47^. We obtained the Cu spectra are nearly identical regardless of different film thicknesses.

On the other hand, the O 1 *s* spectra shows a thickness-dependent shift. The O 1 *s* spectra shown in Fig. 7(b) have two peaks. The high binding peak at ~530.5 eV appears due to surface hydroxides, as reported in bulk XPS results^43,47^. Our main focus is on the primary peak at a lower energy ~528.5 eV that corresponds to the O 1 *s* binding energy spectrum of the LSCO film. We clearly recognize the O 1 *s* peak moves toward less binding energy as film thickness becomes thicker. Figure 7(c) summarizes the binding energy shift of both O 1 *s* and Cu 2*p*3*d*^10^*L*–like peaks relative to the spectra of the 6-nm film.

The shift of core level binding energy Δ*E* (by different film thickness in this case) can be expressed as^48^:

3 $$\Delta E=\Delta \mu +K\Delta Q+\Delta {V}_{M}+\Delta {E}_{R}$$

Δ*μ* represents a change in chemical potential. Since the reference zero of the binding energy is defined as the chemical potential, the binding energy becomes smaller when the chemical potential is lowered. Different from the chemical potential that commonly applies to all the elements of a material in an equilibrium state, specific ions can have different valence states. Δ*Q* stands for the change in valence state. Ions with a higher valence state have a deeper binding energy because fewer electrons screen the nucleus potential. The proportional coefficient *K* means the binding energy shift per unit charge variation. The other two terms of Δ*V*_*M*_ and Δ*E*_*R*_ indicate the changes in the Madelung potential energy and the extra-atomic relaxation energy, respectively. These terms are relatively less important in usual cases.

With this in mind, we interpret the origin of the red shift of the primary oxygen 1 s peak at ~528.5 eV as the film thickness increases. The oxygen spectra need to be understood in an integrated way with the Cu 2*p*3*d*^10^*L*-like spectra that are almost independent of thickness. At this moment, we have two following scenarios.

Scenario 1: Provided that the red shift in O 1 *s* spectra is mainly attributed to the valence state change (Δ*Q* < 0), we are led to the conjecture that hole concentration is reduced as the film thickness increases, i.e. more electrons existent near oxygen ions screen the positive potential from the oxygen nucleus more strongly, thereby the binding energy can become weaker. The almost same Cu 2*p*3*d*^10^*L* spectra, regardless of the different film thicknesses, indicate the valence states of Cu are nearly identical and the chemical potential doesn’t change significantly in all the measured samples. A variation in hole occupancy is more sensitively identified through the oxygen core level XPS.

Scenario 2: Provided that the red shift in O 1 *s* spectra is mainly attributed to the chemical potential change (Δ*μ* < 0), it is deduced that the hole concentration increases as the film thickness increases. Since chemical potential change should be equally applied to the Cu spectra, the nearly identical Cu spectra requires another thickness-dependent effect to compensate the chemical potential change. The increased hole concentration makes the binding energy of Cu 2*p*_3/2_ spectrum be deeper (Δ*Q* > 0). As a result, the two contributions are compensated for each other, leading to negligible changes in the Cu spectra.

The Scenario 2 is more probable in our system because the thicker films show more metallic character as well as the clear superconducting transition approaching the bulk value in the transport measurement. The interfacial regions close to substrate can easily suffer the formation of ionized oxygen vacancies that partially compensate hole carriers and suppress the superconducting state. The similar red shift has been found in LSCO system where the O 1 *s* core level underwent a red shift as Sr doping ratio increases^46,47^. Either O 1 *s* or Cu 2*p*_3/2_ core level, chemical potential shift by ~0.1 eV naively corresponds to a change in Sr doping ratio by Δx ~ 0.15^46,47^. Accordingly, the observed variation is sufficient enough to modify the electronic conduction property. If we choose the Scenario 1, we expect the thinnest sample was overdoped so as to break superconductivity but this seemingly possible scenario doesn’t harmonize with the observed insulating behavior that contradicts the metallic transport behavior usually found in the overdoped region.

## Discussion

Although we have found the spectral signature of electronic state change, only the variation of carrier concentration may not be sufficient to account for all the features of the SIT in LSCO films. We note an abrupt red shift of the oxygen binding energy as much as −0.1 eV at the thickness of 9 nm and the red shift continues up to −0.14 eV at 40 nm (Fig. 7(c)). The abrupt spectral change at 9 nm is not exactly commensurate with the critical thickness (thicker than 9 nm) at the boundary of superconducting and insulating ground states. Moreover, the pronounced peak in *R*_s_ − *T* curve is most likely due to weak localization indicating a presence of microscopic disorders. Random disorders associated with an inhomogeneous presence of the aforementioned oxygen vacancies disturb the fully coherent superconducting state and likely create spatially confined superconducting states incoherent with each other and normal metallic states of unpaired holes. To emerge the supercurrent in the macroscopic transport measurement, the superconducting paths should form through the random spatial mixture of local superconducting states and metallic/insulating states. Even if the disorder density per unit volume is nearly uniform, the disorder effect on transport in the context of percolation theory can be highly dependent on film thickness. If the phase coherent length is close to or greater than film thickness, the electronic conduction might be considered as 2D percolation transport. The percolation threshold of a volume fraction of superconducting states to form the supercurrent paths can depend on the effective spatial dimension determined by the film thickness and disorder-driven coherence length. More detailed theoretical and experimental studies are needed.

## Conclusion

In summary, we explored the thickness-induced SIT in optimally-doped (x = 0.15) LSCO epitaxial thin films that were grown on an almost exactly matched substrate LaAlO_3_. The electronic transport measurements revealed a film-thickness-driven SIT approximately at 10 nm. The resistance versus temperature (*R* − *T*) curves exhibit clear resistance peak feature near superconducting *T*_c_, which could be interpreted by the weak localization. From the experimental XPS studies for the film thicknesses around 10 nm, we also found a red shift of O 1 *s* binding energy as much as −0.14 eV with increasing film thickness whereas the shift of the Cu 2*p*_3/2_ peak was relatively negligible. It is a probable result when more hole carriers are injected into the ligand in thicker samples while the thinner samples have reduced hole carriers because oxygen vacancies locate near the interface.

## Methods

### Materials synthesis

The LSCO epitaxial thin films were grown on LAO substrates by using a pulsed laser deposition (PLD) system equipped with a KrF excimer laser with a wavelength of 247 nm. A PLD target was prepared through the conventional solid-state reaction by mixing La_2_O_3_, SrO, and CuO_2_ powders with the stoichiometric composition ratio at x = 0.15. Before mixing the powders, the La_2_O_3_ powder was baked at 1000 °C for 3 hours to remove humidity. Calcination and sintering processes were performed at 1100 °C for 11 hours, respectively. During film growths, oxygen pressure in the deposition chamber was set to be 100 mTorr and the substrate temperature was 740 °C. The laser fluence and repetition frequency were set to be 0.44 J/cm^2^ and 10 Hz. The typical growth speed was 4 nm per minute. To minimize oxygen vacancy creation, *in-situ* post-annealing processes were performed in an oxygen environment of 500 Torr at 650 °C and 500 °C for 15 min and 10 min, respectively.

### Transport measurement

The transport measurements were performed by using Physical Property Measurement System (PPMS, Quantum Design, Inc.). To avoid the contact resistance issue, we conducted the DC four probe method by using a current source (Keithley 6221) and a nanovoltmeter (Keithley 2182 A). The films were cut into a rectangular shape of approximately 2.5 mm by 5 mm in size. Two indium electrodes were prepared on the 2.5-mm-side edges to supply a constant current. Two additional indium spots in between were made at a gap of ~1 mm to measure the voltage. The *R* − *T* curves were measured with heating at a ramping rate of +3 K/min. Applied DC current was chosen in the range of 1 μA to 100 μA depending on the film thickness to get measurable voltages. In the geometry, the electric fields were applied along an in-plane <100> direction.

### Crystalline structural characterizations

X-ray diffraction was carried out by using X’Pert-PRO MRD (PANalytical). The 2θ − ω scans were measured in the range of 5° to 70° by using 1/4° receiving slit and 1/16° incident slit with an integration time of 1 sec at a step of 0.01°. The RSMs were performed by using with 1/2° receiving slit and 1/8° incident slit. The atomic-resolution cross-sectional scanning TEM images were obtained by Titan Cubed G2 60–300 (FEI) which operated with a double C_S_-corrector and a monochromator. The TEM specimen of a LSCO film was prepared by using focused ion beam (FIB) (Quanta 3D FEG (FEI)). Pt and carbon layers were deposited on the LSCO film to minimize charging effect during FIB etching process. The lift-off was performed with Ga ion beam. Surface topographic images were measured using a scanning probe microscopy (Bruker Multimode V equipped with a Nanoscope controller V) at an ambient condition.

### X-ray photoemission spectroscopy

The XPS was conducted by using K-alpha (Thermo VG scientific) with Al *K*α source at the photon energy of 1486.7 eV. The uncertainty of measurements using the instrument is 50 meV. The beam size on the sample surface was approximately 400 μm in diameter. The x-ray beam was projected onto the film surface at an incident angle of 54.7°. To synchronize the chemical potential of the equipment and the samples, the film surfaces were electrically contacted with the sample stage by metal clips. The sample surfaces were cleaned by *in-situ* Ar ion etching gun (EX06 Ion Source) for 90 seconds before getting spectra.

## Data Availability

The data that support the findings of this study will be available from the corresponding author upon reasonable request.
